# Galnon Facilitates Extinction of Morphine-Conditioned Place Preference but Also Potentiates the Consolidation Process

**DOI:** 10.1371/journal.pone.0076395

**Published:** 2013-10-11

**Authors:** Xiaojie Zhao, Keming Yun, Ronald R. Seese, Zhenyuan Wang

**Affiliations:** 1 Department of Forensic Science, Xi'an Jiaotong University College of Medicine, Xi'an, Shaanxi, PR China; 2 Department of Forensic Medicine, Nanjing Medical University, Nanjing, Jiangsu, PR China; 3 School of Forensic Medicine, Shanxi Medical University, Taiyuan, Shanxi, PR China; 4 Department of Anatomy & Neurobiology, University of California Irvine, School of Medicine, Irvine, California, United States of America; Max Planck Institute of Psychiatry, Germany

## Abstract

Learning and memory systems are intimately involved in drug addiction. Previous studies suggest that galanin, a neuropeptide that binds G-protein coupled receptors, plays essential roles in the encoding of memory. In the present study, we tested the function of galnon, a galanin receptor 1 and 2 agonist, in reward-associated memory, using conditioned place preference (CPP), a widely used paradigm in drug-associated memory. Either before or following CPP-inducing morphine administration, galnon was injected at four different time points to test the effects of galanin activation on different reward-associated memory processes: 15 min before CPP training (acquisition), immediately after CPP training (consolidation), 15 min before the post-conditioning test (retrieval), and multiple injection after post-tests (reconsolidation and extinction). Galnon enhanced consolidation and extinction processes of morphine-induced CPP memory, but the compound had no effect on acquisition, retrieval, or reconsolidation processes. Our findings demonstrate that a galanin receptor 1 and 2 agonist, galnon, may be used as a viable compound to treat drug addiction by facilitating memory extinction process.

## Introduction

Drug addiction, characterized by persistent drug-seeking behaviors, is frequently conceptualized as a disorder of maladaptive memory [1], [2]. Once re-exposed to drug-associated environmental cues, drug-seeking behavior can be reactivated and relapse may occur after years of abstinence. Thus, persistent and unwanted drug-associated memory is believed to be a key contributor to this chronic relapse problem. Many neural systems, including cholinergic, dopaminergic, noradrenergic, serotonergic, glutamatergic, GABAergic, and cannabinoidergic pathways, have been implicated in the formation of drug-associated memory; moreover, many amnestic agents engage these systems, aiming to interrupt aberrant drug-associated memory [3], [4]. Recently, the neuropeptide galanin was suggested to play an important role in addictive behaviors [5], [6]. Galanin is necessary, at least to some degree, for normal learning and memory processes. Intracerebral administration of galanin to rodents prior to training impairs spatial learning and passive avoidance, likely because of galanin's inhibitory effects on acetylcholine transmission within the ventral hippocampus [7]. Moreover, the neuropeptide has been more directly implicated in drug addiction as galanin knockout (KO) mice (GAL −/−) show increased sensitivity to morphine and cocaine but decreased sensitivity to nicotine in the conditioned place preference (CPP) paradigm; locomotor activity is also robustly hyperactive following morphine administration in GAL −/− mice [8]–[10]. These effects may be mediated by galanin's effects on the mesolimbic dopamine system [11].

Galnon, a galanin receptor 1 and 2 agonist, can easily penetrate the blood-brain-barrier and resist enzymatic degradation because of its low molecular weight and lipophilic properties [12]. Moreover, galnon and galanin share many pharmacological and behavioral effects [12], [13]. Indeed, both galanin and galnon lower the maximal seizure score while producing anti-hyperalgesic, antidepressant, and anxiolytic-like effects [12], [14]–[16]. With regards to drug addiction in GAL −/− mice, galnon reverses the morphine-induced increase in locomotor activity, blocks nicotine and cocaine reward in the CPP model, and reduces morphine withdrawal symptoms [8], [9], [17]. In brief, galnon appears to simulate many of galanin's physiological effects, although some other receptors (i.e., NPY1, NK2, M5, and somatostatin) can also be activated by galnon [18].

To integrate these data, and test for a potent therapeutic for drug-addictive behavior, we more specifically studied galnon's role in the different phases of encoding reward-associated memory. Memory processes are generally divided into five phases: acquisition, consolidation, retrieval, reconsolidation, and extinction [19]. In the CPP paradigm, which is based on Pavlovian classical conditioning, these memory processes can be easily identified and manipulated. In the present series of experiments, the effects of galanin activation on morphine-induced CPP memories were evaluated by administing galnon at different time points and phases of the CPP paradigm.

## Materials and Methods

### Animals and drugs

C57BL/6J male mice (8 weeks old) weighing 20–25 g were obtained from Beijing Vital River Laboratories. They were housed in groups of four under constant temperature (23±2°C) and maintained on a 12 hour light/dark cycle (lights on at 7 a.m.). Food and water was available *ad libitum*. All mice were handled individually and sham injected intraperitoneally (i.p.) once daily for a week. After one week of habituation, 9 week old mice were used for the further experiments. The experimental protocols (Permit Number: 200910011) were approved by the Xi'an Jiaotong University Laboratory Animal Administration Committee and performed according to the Xi'an Jiaotong University Guidelines for Animal Experimentation and also conformed to the Guide for the Care and Use of Laboratory Animals published by the US National Institutes of Health.

Morphine hydrochloride (Mor) was purchased from Qinghai Pharmaceutical Group and dissolved in sterile 0.9% saline. Galnon (CAS: 475115–35–6, Fmoc-b-Cha-Lys-AMC) was synthesized by Shanghai Hanhong Chemical Limited Company. Galnon was initially dissolved in 100% DMSO, then diluted in 0.9% saline and briefly sonicated to obtain a uniform solution. In the final solution, the concentration of DMSO was not more than 1%, which has been previously demonstrated to not affect animal behavior [20]. All dissolved drugs were given by i.p. injections at a volume of 10 ml/kg.

### CPP procedures and locomotor activity

The CPP chambers consisted of two identical wooden compartments (white and black; 15×15×37 cm) with different visual and tactile cues. White walls with a stainless-steel mesh floor and black walls with a stainless-steel bar floor (W and B compartments, respectively) were separated by a sliding wood panel with a 5 cm×7 cm door in the center of the base. The door remained open during the test phase, but was shut during the training phase to prevent movement of mice between two compartments. Night vision equipped cameras were used to digitally record the behavior of mice; time spent in the two compartments and distance traveled was measured by Shanghai Jiliang software.

The CPP paradigm is divided into three phases. In the *pre-conditioning test* (pre-test), mice are allowed to move freely between the two compartments for 15 min to determine any baseline preferences for the W or B compartment prior to morphine administration. During the *training period*, mice were treated once daily for six consecutive days with three cycles of alternating i.p. injections of morphine in compartment W and then saline in compartment B. Immediately following each injection, mice were immediately confined for 40 min to either compartment W or B compartment for morphine or saline injections, respectively. In the control group, saline was given every training day, irrespective of compartment. The *post-conditioning test* (post-test) was given 24 h following all three training cycles. Like the pre-test, the door separating the B and W compartments was left open and mice freely moved 15 min in the post-test. All compartments were cleaned and wiped dry between animal runs. The counterbalanced design for day of treatment was used: half of the mice were injected morphine before they were placed in white walls with a stainless-steel mesh floor compartment, and half received saline prior to placement in the black walls with a stainless-steel bar floor compartment in the same day. The time spent in the white compartment (conditioned stimulus, CS+) during the post-conditioning test minus the time in the CS+ during the pre-conditioning test, assigned as “Post CS+ minus Pre CS+”, was used as an index of preference [21]. A positive CPP effect is observed when the morphine treatment group exhibits a significantly higher value as compared to the control group (only saline treatment).

Locomotor activity was measured as the total distance traveled in a separate chamber (43×43×43 cm) for 1 h using the automated Smart system (Panlab, Barcelona, Spain).

### Experimental design

#### Experiment 1: Acquisition

Before testing the effect of galnon on the acquisition of CPP memories, two possibilities must be excluded: 1.) high doses galnon affect locomotor activity of mice; 2.) galnon itself might induce CPP, independent of morphine administration. To test the former, two doses of galnon (5 and 10 mg/kg, i.p.) were injected 15 min before mice were placed into the locomotor chambers for 1 hr. Automated systems measured the total distance traveled. The dose that did not affect total distance traveled was used to test the effects of galanin activation on CPP and CPA formation. Much like the morphine experiments, this control study was performed by confining animals to a chamber for 40 min following galnon injection during training days.

**Figure 1:**
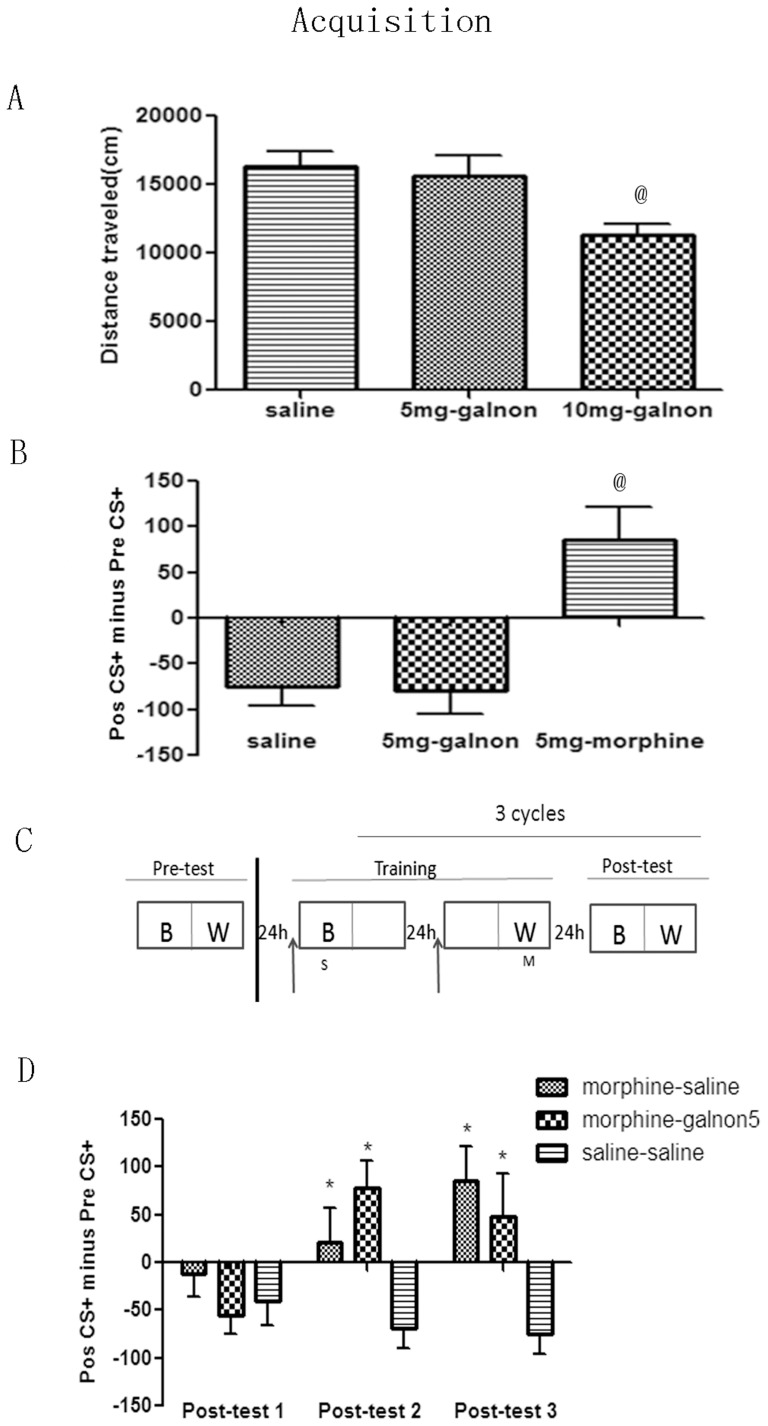
Effect of galnon on acquisition of morphine-induced CPP. A, 10/kg galnon injection impaired the locomotor activity. B, Galnon itself had no effects of CPP and CPA. C, Behavioral procedure for injection time points in acquisition process. Upward arrows indicate galnon or saline injection. D, The acquisition process of morphine-induced CPP did not be influenced by galnon. ^@^ *p*<0.05 vs the other groups; * *p*<0.05 vs saline-saline group in each test.

To more directly test if galanin activation affects the acquisition of morphine-induced CPP, galnon or saline was administered 15 min before morphine or saline was injected on each of the six training days (morphine-galnon and morphine-saline groups) (Fig. 1C). Following the completion of each training cycle, animals underwent a post-conditioning test as described above and the “Post CS+ minus Pre CS+” was quantified. In a separate control group (saline-saline), saline was injected to mice 15 min before all training phase where saline was given every training day, irrespective of compartment.

#### Experiment 2: Consolidation

**Figure 2:**
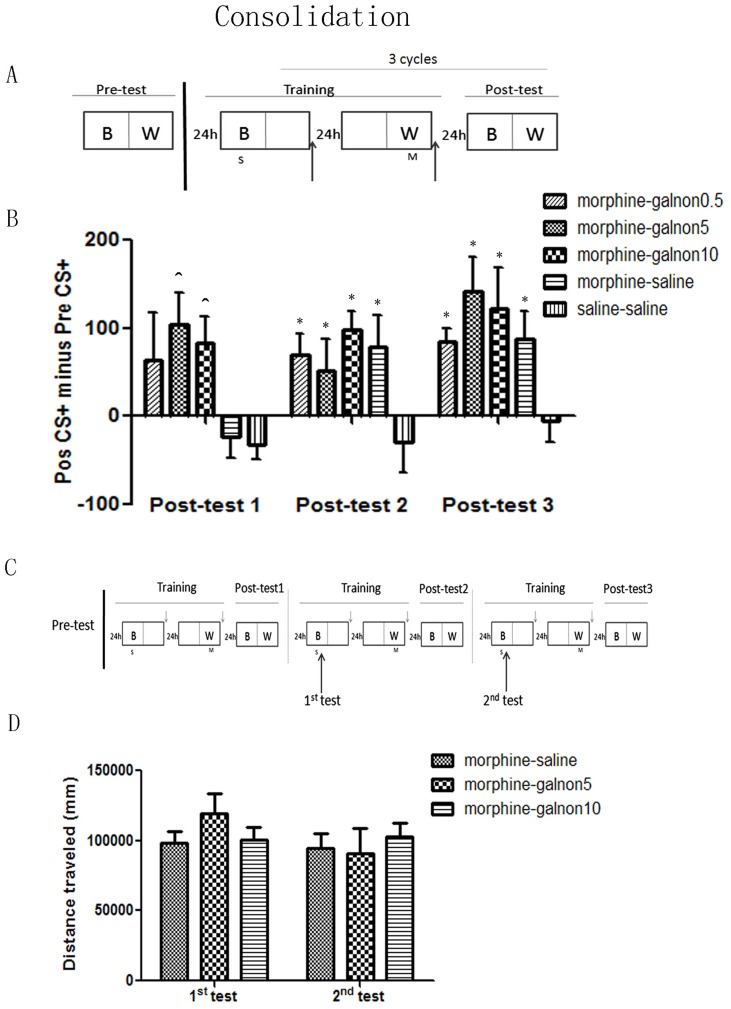
Effect of galnon on consolidation of morphine-induced CPP. A, Behavioral procedure for injection time points in consolidation process. Upward arrows indicates galnon or saline injection. B, Galnon enhanced the consolidation process of morphine-induced CPP. C, Behavioral procedure for time points of locomotor activity tests after post-training injection of 10 mg/kg galnon. Down arrows indicate galnon or saline injection. D, The locomotor activity in two training days did not be influenced by post-training injection of 10 mg/kg galnon. ^∧^ *p*<0.05 vs morphine-saline group; * *p*<0.05 vs saline-saline group in each test.

To test if galnon affects the consolidation of morphine-induced CPP, different groups of mice received saline or different doses of galnon (0.5, 5, 10 mg/kg) immediately after each training day (Fig. 2A). A post-test was performed as described above after each training cycle to determine whether place preference is formed.

The studies in experiment 1 tested if 10 mg/kg galnon acutely affected locomotion 15 min after the compound was injected. Here, the distances traveled were recorded twice in morphine-galnon5, morphine-galnon10 and morphine-saline group (control group) to assess how prior galnon treatment can affect activity as measured 24 h later; two tests were performed immediately after injection of saline for 40 min in compartment B (in the last two saline conditioning sessions) (Fig. 2C).

#### Experiment 3: Retrieval

**Figure 3:**
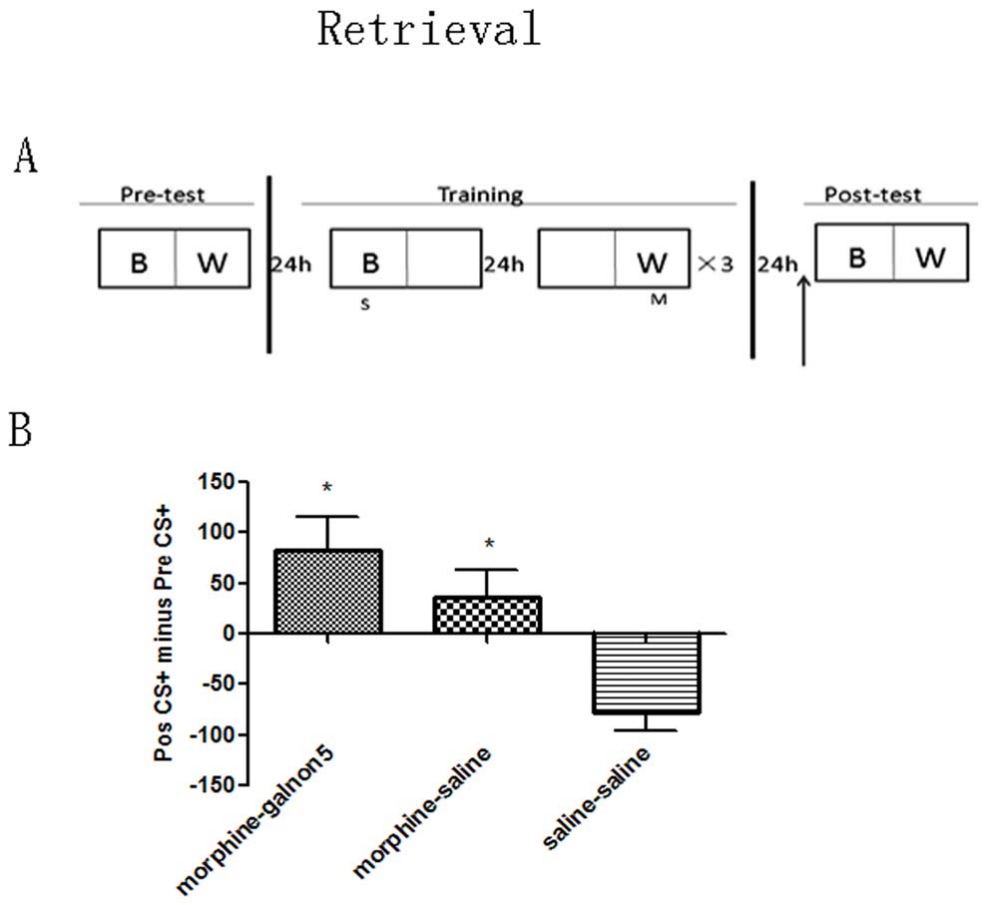
Effect of galnon on retrieval of morphine-induced CPP. A, Behavioral procedure for injection time points in retrieval process. Upward arrows indicate galnon or saline injection. B, Galnon did not affect retrieval process of morphine-induced CPP. * *p*<0.05 vs saline-saline group in each test.

To test if galanin activation affected retrieval of morphine-induced CPP, galnon (5 mg/kg) or saline was given 15 min before the post-test that followed the third completed training cycle (Fig. 3A).

#### Experiment 4: multiple post-retrieval injections

**Figure 4:**
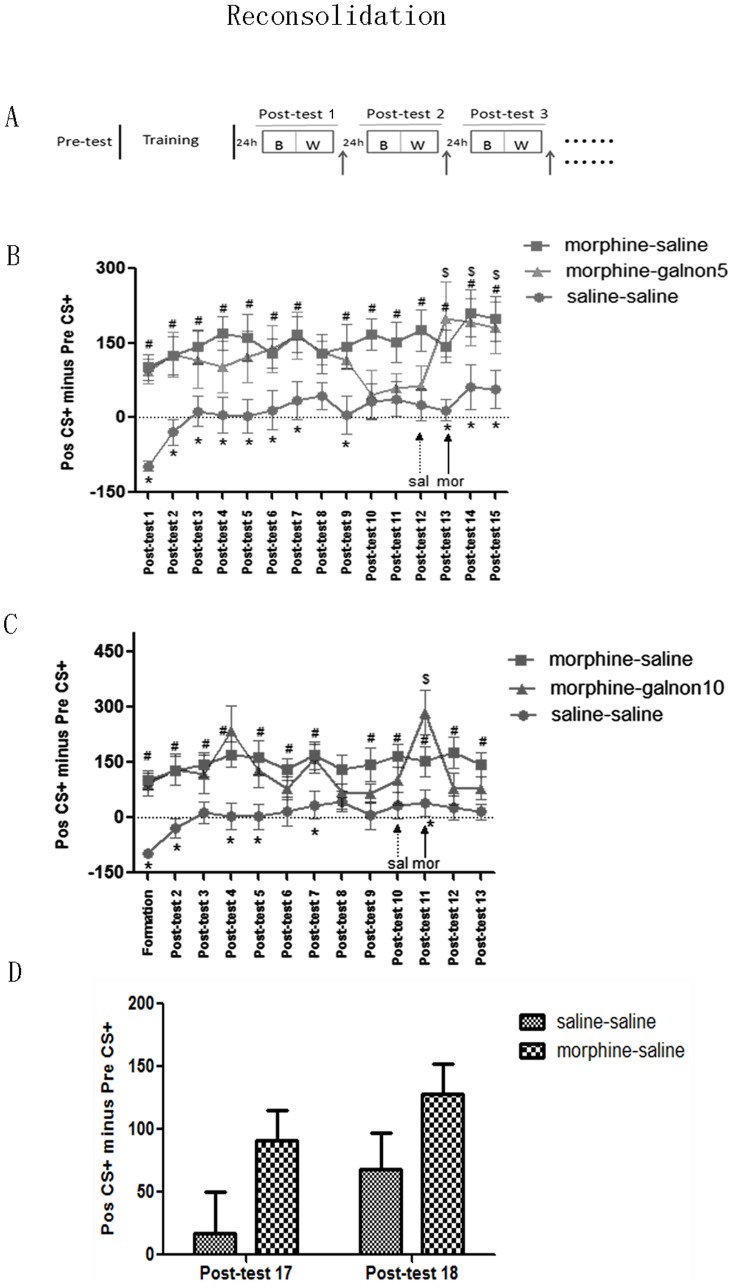
Effect of multiple post-retrieval galnon injections on expression of morphine-induced CPP. A, Behavioral procedure for mutiple post-retrieval galnon injections. Upward arrows indicate galnon or saline injection. B & C, Galnon facilitated CPP extinction, but did not impair the reinstatement of morphine priming, though 10 mg/kg galnon had stronger effect for the quickly recovering and maintaining of extinction-effect after priming. D, The time points of the disappearing CPP-effect in morphine-saline group. # *p*<0.05 morphine-saline vs saline-saline, * *p*<0.05 morphine-galnon5 or morphine-galnon10 vs saline-saline, $ *p*<0.05 vs saline (sal) priming day test.

Two doses of galnon (5 and 10 mg/kg) were used in these studies, which tested if galanin activation influenced reconsolidation and extinction of morphine-induced CPP memories. When all the training cycles were completed, daily post-conditioning tests, which are similar to the pre-test (mice are allowed to move freely among the two compartments for 15 min), were performed with galnon or saline injected immediately following each post-test (Fig. 4A). These post-tests and injections continued until CPP expression was extinguished over two consecutive days by galnon treatment, as determined by a disappearance of statistical difference from the saline-saline group. At this point, galnon groups were given saline and morphine (3 mg/kg) injections immediately before each post-test over the next two respective days to test if CPP can be reinstated.

### Data analysis

One-way analysis of variance (ANOVA) was performed in retrieval experiment and the experiments to determine the effect of galnon on locomotor activity and whether galnon itself had CPP or CPA effects (Fig. 1A & B). Two-way analysis of variance (ANOVA) for repeated-measure (treatment × test day) was used in the other experiments. Fischer's least significant difference (LSD) was used as the post-hoc test for ANOVAs.

## Results

### Effect of galnon on acquisition of morphine-induced conditioned place preference

As shown in Fig. 1A, mice injected with 10 mg/kg of galnon traveled less total distance than vehicle-treated mice (F_(2,28)_ = 5.395, *p* = 0.011; saline (n = 10) vs 5 mg-galnon (n = 9), *p* = 0.696; saline vs 10 mg-galnon (n = 10), *p* = 0.005). As such, we chose 5 mg/kg galnon as a suitable dose to test if galanin activation itself influences the proper expression of CPP. In the absence of morphine, expression of CPP in animals injected with 5 mg/kg galnon or saline did not differ (F_(2,23)_ = 5.395, *p*<0.001; 5 mg-galnon (n = 8) vs saline (n = 8), *p* = 0.908), which also suggested that galnon itself did not have CPP or CPA effects. However, the degree of CPP induced by morphine was significantly different than that in animals injected with either galnon or saline (5 mg-galnon vs 5 mg-morphine (n = 8), *p*<0.001; saline vs 5 mg-morphine, *p* = 0.001; Fig. 1B).

To test if galanin activation affects acquisition of morphine-induced CPP memory, galnon was administered 15 min before each training day's morphine or saline injection (Fig. 1C). Fig. 1D illustrates that after two training cycles, CPP expression was robust in groups that received either saline or 5 mg/kg galnon 15 min prior to morphine training (treatment: F_(2, 22)_ = 6.450, *p* = 0.006; test day: F_(2, 44)_ = 2.888, *p* = 0.066; treatment × test day: F_(4, 44)_ = 5.710, *p* = 0.001. *Post hoc analysis*: Post-test 2: F_(2,22)_ = 9.468, *p* = 0.001; saline-saline (n = 8) vs all others *p*<0.01; and Post-test 3: F_(2,22)_ = 5.111, *p* = 0.015; saline-saline vs others all *p*<0.05). Moreover, 5 mg/kg galnon did not significantly affect morphine-induced CPP effect (Post-test 2: morphine-galnon5 (n = 9) vs morphine-saline (n = 8), *p* = 0.197; Post-test 3: morphine-saline vs morphine-galnon5, *p* = 0.470). Collectively, these results demonstrate that galnon has no effect on the acquisition of morphine-induced CPP.

### Effect of galnon on consolidation of morphine-induced conditioned place preference

Galnon was administered at three doses (0.5, 5, 10 mg/kg) immediately following training episodes to test if galanin activation influences the consolidation phase of morphine-induced CPP. All mice underwent three cycles of conditioning to specific chambers with morphine and saline (Fig. 2A). Two different doses of galnon (5 mg/kg, n = 9; 10 mg/kg, n = 9) induced robust CPP on the first post-test; saline-injected mice, irrespective of morphine pre-treatment, did not exhibit any preference for the conditioned compartment; this trend was also seen in the lowest dose galnon group (0.5 mg/kg, n = 9), though this effect did not reach statistical significance (treatment: F_(4, 39)_ = 4.118, *p* = 0.007; test day: F_(2, 78)_ = 5.582, *p* = 0.007; treatment × test day: F_(8, 78)_ = 2.214, *p* = 0.035. *Post hoc analysis*: Post-test 1: F_(4,39)_ = 3.034, *p* = 0.029; saline-saline (n = 8) vs morphine-saline (n = 9), morphine-galnon0.5, morphine-galnon5, morphine-galnon10, *p* = 0.870, 0.083, 0.023, 0.039; Fig. 2B).

Similar to the results in experiment 1 (see Fig. 1D), the CPP effect was evident in the morphine-saline group after 4 days (2 cycles) training, as compared to the control group (saline-saline) (Post-test 2: F_(4,39)_ = 3.056, *p* = 0.028; saline-saline vs morphine-saline, *p* = 0.021. Post-test 3: F_(4,39)_ = 4.260, *p* = 0.006; saline-saline vs morphine-saline, *p* = 0.044; Fig. 2B). In contrast to what was observed with a single injection of high dose galnon, consecutive post-training injections of 10 mg/kg galnon (see Fig. 2C) did not influence the total distance traveled on the later training days (treatment: F_(2, 28)_ = 0.031, *p* = 0.970; treatment × test day: F_(2, 28)_ = 2.392, *p* = 0.110; Fig. 2D).

As such, galnon promotes the consolidation process of morphine-induced CPP without interfering with underlying locomotor function.

### Effect of galnon on retrieval of morphine-induced conditioned place preference

To test if galanin activation affects the retrieval of CPP memory, animals were injected with 5 mg/kg galnon 15 min before the post-test that followed the third completed training cycle (Fig. 3A). As illustrated in Fig. 3B, 5 mg/kg galnon injected at this time did not affect the retrieval of drug-associated CPP memories (F_(2, 30)_ = 7.784, *p* = 0.002; morphine-galnon5 (n = 12) vs morphine-saline (n = 10), *p* = 0.260), and all morphine treatment groups expressed the CPP effect (saline-saline (n = 9) vs morphine-galnon5, morphine-saline, p = 0.001, 0.017).

### Effect of multiple post-retrieval galnon injections on expression of morphine-induced conditioned place preference

As shown in Fig. 4B, the morphine-galnon5 group ceased to express a statistical difference in “Post CS+ minus Pre CS+” values from the saline-saline group over two consecutive days starting at Post-test 10 (treatment: F_(2, 26)_ = 6.658, *p* = 0.005; test day: F_(14, 364)_ = 3.571, *p*<0.001; treatment × test day: F_(28, 364)_ = 1.542, *p* = 0.046. *Post hoc analysis*: Post-test 10: F_(2, 26)_ = 3.726, *p* = 0.038; saline-saline(n = 10) vs morphine-galnon5 (n = 9), morphine-saline (n = 10), *p* = 0.602, 0.015; and Post-test 11: F_(2, 26)_ = 2.976, *p* = 0.069). This data suggests that galanin also promotes the extinction of morphine-induced CPP.

Immediately prior to Post-test 13, 3 mg/kg of morphine was injected to test if extinction of CPP could be reinstated; morphine induced a robust and immediate rescue of CPP (F_(2, 26)_ = 5.481, *p* = 0.010; saline-saline vs morphine-galnon5, morphine-saline, *p* = 0.003, 0.045). This rescue was not transient, as it persisted through Post-test 14 and Post-test 15 (Post-test 14: F_(2, 26)_ = 3.771, *p* = 0.036; saline-saline vs morphine-galnon5, morphine-saline, *p* = 0.014, 0.048. Post-test 15: F_(2, 26)_ = 3.464, *p* = 0.046; saline-saline vs morphine-galnon5, morphine-saline, *p* = 0.035, 0.036). Additionally, this rescue appeared to require morphine as it was not present on the day prior, Post-test 12, when saline was injected immediately prior to the post-test (F_(2, 26)_ = 3.442, *p* = 0.048; saline-saline vs morphine-galnon5, morphine-saline, *p* = 0.102, 0.016).

With higher doses of galnon (10 mg/kg), CPP was extinguished two days earlier than the 5 mg/kg group (treatment: F_(2, 27)_ = 6.526, *p* = 0.005; test day: F_(12, 324)_ = 4.209, *p*<0.001; treatment × test day: F_(24, 324)_ = 2.247, *p* = 0.001. *Post hoc analysis*: Post-test 8: F_(2, 27)_ = 1.251, *p* = 0.302; and Post-test-9: F_(2, 27)_ = 3.709, *p* = 0.038; saline-saline vs morphine-galnon10 (n = 10), morphine-saline, *p* = 0.235, 0.011). CPP was also reinstated by 3 mg/kg morphine; however, this CPP rescue with morphine did not persist in the days following repriming (Post-test 10: F_(2, 27)_ = 2.706, *p* = 0.145; saline-saline vs morphine-galnon10, morphine-saline, *p* = 0.306, 0.044. post-test-11: F_(2, 27)_ = 6.862, *p* = 0.004; saline-saline vs morphine-galnon10, morphine-saline, *p* = 0.001, 0.047. Post-test 12: F_(2, 27)_ = 3.848, *p* = 0.034; saline-saline vs morphine-galnon10, morphine-saline, *p* = 0.363, 0.011. Post-test 13: F_(2, 27)_ = 4.874, *p* = 0.016; saline-saline vs morphine-galnon10, morphine-saline, *p* = 0.131, 0.004).

As shown in Fig. 4D, the CPP effect of the morphine-saline group was not statistically different than the saline-saline group starting at Post-test 17 (treatment: F_(1, 18)_ = 3.233, *p* = 0.089; treatment × test day: F_(1, 18)_ = 0.317, *p* = 0.581).

## Discussion

The present study examined the effect of galnon, a galanin receptor 1 and 2 agonist, on morphine-induced reward memory using the CPP paradigm. Our results directly show that galnon enhances the consolidation process of morphine-induced CPP without affecting either the acquisition or retrieval processes.

Many studies have demonstrated that the galanin system mediates learning and memory processes. For example, galanin is over-expressed in Alzheimer's disease, administration of galanin interferes with proper consolidation of several learning and memory tasks, and galanin transgenic mice exhibit marked deficits in the Morris water maze (see review [22]). Further investigations show that the inhibitory action of galanin on cholinergic transmission may be intimately involved in these learning and memory deficits [23]. While most studies demonstrate that galanin plays an inhibitory role in learning and memory processes, there is some evidence that this is not always the case. For example, galanin knockout mice have no deficit in single-item object recognition memory [24]. Moreover, our results show that galnon, a galanin receptor 1 and 2 agonist, enhances the consolidation of morphine-induced CPP memory. Differences in animal models, recruited brain regions, and injection paradigms may help account for the discrepancies in the literature. In some previous research, mice were also pretreated with galnon similar to our “acquisition” protocol, with the compound impairing the CPP effect induced by cocaine or nicotine [9], [25]. However, in their protocols galnon is given only before addictive drugs conditioning which is different to our “acquisition” protocol where galnon is injected to mice before both saline and morphine conditioning; these different injection paradigms may result the discrepancies. And, this interpretation is confirmed by a additional experiment (unpublished data) where we used another addictive drug–cocaine (3 mg/kg) to replace morphine in “*Experiment 1: Acquisition*”, and got a similar conclusion–galnon can not influence the acquisition process of CPP. One possibility is that galnon may partially counteract the effects of the addictive drug in a protocol that did not inject galnon before saline conditioning; if galnon was also given before saline conditioning, as it is the case in the present work, then galnon similarly affected both training processes. In addition, the galanin receptor 1 exhibits mostly opposite behavioral effects as compared to the galanin receptor 2 receptor [26] may also be another reason for the discrepancies.

What is the role of galnon in the reconsolidation and extinction of morphine-induced CPP? Extinction is frequently considered a new form of memory encoding that inhibits or overrides the initial learning episodes rather than erasing them. Moreover, the extinguished memory may be recovered by reinstatement (i.e., in this case, via morphine administration) [27], [28]. Reconsolidation, on the other hand, is proposed to update memories by making the existing memory trace more labile such that the encoding of existing memory trace can be further strengthened by additional training or interrupted by some pharmacological agents [29]. Animals in experiment 4 received daily post-test injections of different doses of galnon (5 and 10 mg/kg). Low dose (5 mg/kg) and high dose (10 mg/kg) galnon quickly extinguished the CPP effect, though the high dose group reached the extinction criterion faster than low dose group. Administration of 3 mg/kg morphine following extinction, however, reinstated the CPP effect. These data indicate that the new memory trace in which a given context is no longer associated with any specific reward, such that it is extinguished, can indeed be accelerated by galnon; however, the original memory of morphine-induced CPP is not erased, as it can be reinstated following morphine priming. Moreover, in experiment 2, the consolidation process was facilitated which implies that galnon can accelerate new memory formation, give more reason to interpret the phenomena from extinction perspective. While at the same time, galnon may have no effect on the reconsolidation process.

However, for the reconsolidation process, there is a possibility that post-test trial employed in experiment 4 is not strong enough to reactivate the original memory and thus reconsolidation fails. For examples, some investigators demonstrated that an established morphine-induced CPP could be interrupted by anisomycin or cycloheximide (protein synthesis inhibitors) when animals experience the conditioned context and the drug, rather than only after contextual recall [30], which implicates that only contextual recall can not successfully induce the reconsolidation process. Others also argue that the more similar the reactivation and training sessions are, the easier it is to induce reconsolidation [31], [32]. However, there is no evidence to rule out the possibility that the reinforcer itself (e.g., morphine) will also influence memory processes [33]–[38] and may then confound the efficacy of drug being tested (e.g., galnon). Also, reconsolidation has been successfully elicited when the reactivation trial lacks the reinforcer (i.e., only the CS present) (see review [39]). Moreover, two studies [40], [41] using the same drug-associated memory paradigm that we employed also demonstrated that the same reactivation procedures successfully blocked reconsolidation. Another possibility is that excessive training cycles or high doses of morphine make the proper intervention effect of galnon on reconsolidation more difficult. For example, in mice that receive three footshocks, reconsolidation is much more resistant than in those receiving only one footshock [42]. However, and importantly, several previous studies confirmed that three training cycles and the dose of 5 mg/kg morphine used in the CPP protocol here does not make the intervention effect of galnon on reconsolidation difficult to achieve [31], [43]–[45]. Another group showed that reconsolidation trace is dominant following intensive training paradigms, but not a single training trial [46].

Based on the above analysis, we conclude that galnon has no effect on the reconsolidation process but does facilitate the extinction process of morphine-induced CPP. Because only one extinction training paradigm was used in our experiment, more research is needed to confirm the current positive findings using other training methods of extinction, such as longer training procedure (similar to acquisition training, but mice only received saline [47], [48]).

In summary, our work illustrates that galnon, a galanin receptor 1 and 2 agonist, enhances consolidation and extinction of morphine-induced CPP memory. Curiously, however, galnon had no effect on acquisition, retrieval, or reconsolidation processes. The facilitated effect of galnon on extinction may be of potential value in the treatment of drug addiction.
